# Developing a model Fracture Liaison Service consultation with patients, carers and clinicians: a Delphi survey to inform content of the iFraP complex consultation intervention

**DOI:** 10.1007/s11657-021-00913-w

**Published:** 2021-03-24

**Authors:** Laurna Bullock, Fay Crawford-Manning, Elizabeth Cottrell, Jane Fleming, Sarah Leyland, John Edwards, Emma M Clark, Simon Thomas, Stephen Chapman, Christopher Gidlow, Cynthia P Iglesias, Joanne Protheroe, Robert Horne, Terence W O’Neill, Christian Mallen, Clare Jinks, Zoe Paskins

**Affiliations:** 1 School of Medicine, Keele University, Newcastle, Staffordshire UK; 2 Haywood Academic Rheumatology Centre, Midlands Partnership NHS Foundation Trust, Stoke-on-Trent, Staffordshire UK; 3 Cambridge Public Health, University of Cambridge & Addenbrooke’s Hospital Fracture Liaison Service, Cambridge University Hospitals NHS Trust, Cambridge, UK; 4 Royal Osteoporosis Society, Bath, UK; 5 Bristol Medical School, Faculty of Health Sciences,, University of Bristol, Bristol, UK; 6 School of Pharmacy and Bioengineering, Keele University, Newcastle, Staffordshire UK; 7 Centre for Health and Development, Staffordshire University, Stoke-on-Trent, Staffordshire UK; 8 Department of Health Sciences, University of York, York, UK; 9 Danish Centre for Healthcare Improvement (CHI), Aalborg University, Aalborg, Denmark; 10 Centre for Behavioural Medicine, UCL School of Pharmacy, University College London, London, UK; 11 Centre for Epidemiology Versus Arthritis, University of Manchester & NIHR Manchester Biomedical Research Centre, Manchester University NHS Foundation Trust, Manchester, UK

**Keywords:** Osteoporosis, Fracture Liaison Services, Delphi survey, Intervention development, Consultation, iFraP

## Abstract

**Summary:**

Fracture Liaison Services are recommended to deliver best practice in secondary fracture prevention. This modified Delphi survey, as part of the iFraP (Improving uptake of Fracture Prevention drug Treatments) study, provides consensus regarding tasks for clinicians in a model Fracture Liaison Service consultation.

**Purpose:**

The clinical consultation is of pivotal importance in addressing barriers to treatment adherence. The aim of this study was to agree to the content of the ‘model Fracture Liaison Service (FLS) consultation’ within the iFraP (Improving uptake of Fracture Prevention drug Treatments) study.

**Methods:**

A Delphi survey was co-designed with patients and clinical stakeholders using an evidence synthesis of current guidelines and content from frameworks and theories of shared decision-making, communication and medicine adherence. Patients with osteoporosis and/or fragility fractures, their carers, FLS clinicians and osteoporosis specialists were sent three rounds of the Delphi survey. Participants were presented with potential consultation content and asked to rate their perception of the importance of each statement on a 5-point Likert scale and to suggest new statements (Round 1). Lowest rated statements were removed or amended after Rounds 1 and 2. In Round 3, participants were asked whether each statement was ‘essential’ and percentage agreement calculated; the study team subsequently determined the threshold for essential content.

**Results:**

Seventy-two, 49 and 52 patients, carers and clinicians responded to Rounds 1, 2 and 3 respectively. One hundred twenty-two statements were considered. By Round 3, consensus was reached, with 81 statements deemed essential within FLS consultations, relating to greeting/introductions; gathering information; considering therapeutic options; eliciting patient perceptions; establishing shared decision-making preferences; sharing information about osteoporosis and treatments; checking understanding/summarising; and signposting next steps.

**Conclusions:**

This Delphi consensus exercise has summarised for the first time patient/carer and clinician consensus regarding clearly defined tasks for clinicians in a model FLS consultation.

**Supplementary Information:**

The online version contains supplementary material available at 10.1007/s11657-021-00913-w.

## Introduction

In the UK, Fracture Liaison Services (FLSs) are recommended to deliver best practice in secondary fracture prevention [1]. These services are typically Allied Health Professional (AHP) or nurse-led and systematically identify patients aged 50 years and over who have experienced a fragility fracture [2]. Evidence-based medications include bisphosphonates, as recommended by National Institute for Health and Care Excellence (NICE) as the first-line therapy for patients with osteoporosis (underlying bone fragility) and/or high fracture risk [3]. Bisphosphonates are inexpensive, cost-effective and readily available and reduce fracture risk by 20–70% (dependent on fracture site) [3].

Clinical Standards for FLSs [4] comprise four aspects of care in the patient pathway: identification, investigation, providing information and support and intervention. These standards recommend follow-up to support adherence to treatment, as bisphosphonate therapy adherence is known to be low and limits treatment effectiveness. National audit results, which monitor performance against these UK standards, indicate that when bisphosphonates are recommended in FLSs, long-term patient adherence is no better than in non-specialist settings and possibly worse. 2018 data indicates that of those patients in whom FLSs recommended starting treatment, only 23% remained adherent at 12 months [2].

Low adherence to osteoporosis treatment globally and the high rate of consequent fragility fractures have been described as the ‘osteoporosis crisis’ [5, 6]. Key contributing factors to low adherence rates include patient (and primary care clinicians’) concerns about harms, uncertainty about treatment benefits and lack of clarity about what constitutes treatment success [7]. This highlights the importance of clinicians understanding and using relevant evidence in the clinical consultation to clearly communicate risks. Clinicians need to feel confident discussing risks of poor disease outcome (prognosis) and the harms and benefits relating to treatment options and facilitating patients to articulate their values and preferences to promote informed decision-making [8]. The Royal (previously National) Osteoporosis Society (ROS) recommends the provision of information as a core component of management [9]. Evidence across a range of conditions and contexts demonstrates that effective use of information within a consultation can increase patient satisfaction, facilitate participation in the consultation and promote trust [8]. Importantly, evidence also suggests that effective use of communication may improve patients’ treatment initiation rates [10] and strengthen their commitment to preventative treatments [11].

The iFraP study (improving uptake of Fracture Prevention drug treatment) aims to develop and evaluate a theoretically informed complex consultation intervention to facilitate informed decision-making about fracture prevention treatment, with a long-term aim of improving informed medication adherence [12]. iFraP includes a computerised decision support tool (CDST) and clinician training package to support clinical decision-making and patient-clinician communication about the patient’s diagnosis, medication options (e.g. bisphosphonates) and lifestyle management for bone health (e.g. smoking, physical activity).

This paper reports findings from a modified Delphi survey with patients, carers and clinicians that aimed to agree the content of the iFraP ‘model FLS consultation’. This study took place in the context of the iFraP intervention development (iFraP-D) study designed to define the content and theoretical basis for the iFraP intervention [12].

## Methods

### Co-design of the Delphi survey

**Fig. 1:**
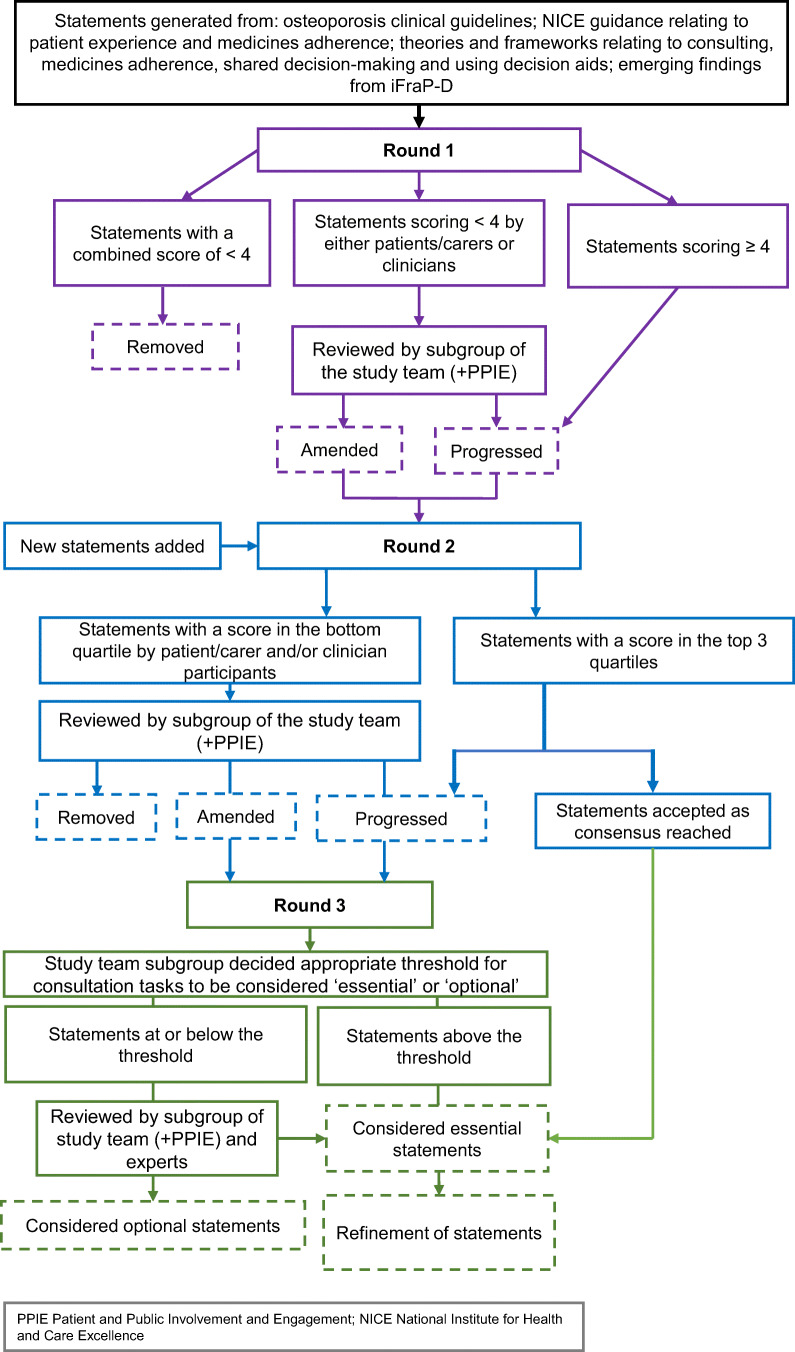
Overview of Delphi methods

A modified, three-round, online Delphi survey (Fig. 1) [13] was co-designed in partnership with members of the iFraP stakeholder group and patient advisory group (PAG), with the aim to develop a survey with a common, understandable language for patients, carers and clinicians. The stakeholders included FLS nurses, pharmacists, GPs, osteoporosis specialists (representing elderly care, rheumatology and metabolic bone medicine) and representatives from the ROS and Health Literacy UK, supported by a behaviour change expert, with expertise in medicine adherence. Patient representation was a key element to ensure the final intervention remained targeted at patient priorities. Six (4:2 female/male) patients with experience of osteoporosis and/or fractures (supported by a Patient and Patient Involvement and Engagement (PPIE) support worker) attended a dedicated PAG, with two PAG members attending stakeholder meetings.

Two stakeholder meetings were conducted to interpret the findings of the evidence synthesis (see Statement generation) in the context of FLS and discuss example explanations, which formed the basis of statement wording. The PAG contributed to the design of the study, including (i) how best to recruit and explain the Delphi survey to patients/carers; (ii) design of the survey, by developing clinical vignettes and (re)wording statements; and (iii) and piloting. Two PAG members also attended survey analysis and interpretation meetings (Rounds 1 and 2) to consider whether statements should be removed or be amended and helped to reword statements (see Data collection and analysis). To promote transparency and address hypothetical concerns of bias, there was clear documentation of the statements before and after each time-point at which the PAG provided input so that the influence of the PAG was recorded and reported clearly. Furthermore, discussion with the PAG on statement inclusion (in conjunction with a clinician and 2 social scientists) was informed not only by PAG views but also underpinning theory, other emergent findings in iFraP-D (e.g. focus groups), explanatory Delphi free-text comments, whether the item was scored low by patients/carers or clinicians and responses to other similar items in the Delphi.

### Delphi participants and recruitment

Delphi surveys aim to recruit expert participants [14]. In the context of FLS consultations, experts included:

1. Patients with osteoporosis and/or fragility fractures and/or their carers recruited via the ROS supporters network
2. Clinicians with experience of consulting with patients, where fracture risk is calculated and fracture prevention treatments are recommended (e.g. endocrinologists, rheumatologists and FLS clinicians), recruited via ROS healthcare professional mailing lists and the study team’s clinical networks

### Statement generation

The Delphi approach is described as ‘modified’ as in the first round participants were presented with a list of statements to consider rather than generating their own list. However, participants had the opportunity to suggest additional consultation tasks.

Each ‘statement’ corresponded to a clinician task for the consultation and included clinician decision-making tasks and considerations, eliciting information, giving information, example explanations and hypothetical use of the CDST. Statements were generated from a number of sources, including:

1. Clinical guidelines for the assessment and management of osteoporosis/fragility fractures that refer to tasks for the clinician in the consultation [3, 15–25].
2. NICE guidance (not specific to osteoporosis) relating to the conduct of the consultation, in enhancing patient experience and medicine adherence [26, 27].
3. Theories and frameworks relating to consulting, medicine adherence, shared decision-making and using decision aids [28–30].
4. Emerging findings from iFraP-D, i.e. stakeholder and PAG discussions, and qualitative findings from focus groups. Additionally, Delphi free-text comments provided by participants in Round 1 generated additional statements for inclusion in Round 2.

Statements derived from UK and European clinical guidelines were sourced from an evidence synthesis exercise which included a systematic search, quality appraisal and data extraction process (conducted March–June 2019, more details in Supplementary Material 1). These statements were used in Round 1. Statements derived from theory, frameworks and emerging findings, fed into both Rounds 1 and 2.

Extracted statements from the evidence synthesis were organised into stages of the consultation. To this end, a framework for the consultation was developed, informed by the phases identified in the evidence synthesis, and underpinning theory, frameworks of shared decision-making and principles of health literacy [28, 30–32]. The framework included a series of stages and tasks for the clinician to progress through during the consultation, including gathering information, considering therapeutic options and eliciting patient knowledge, through to summarising and signposting (Table 1).

**Table 1:** Example statements for each consultation stage

Consultation stage/clinician task	Example statement
1. Greeting/introduction	The clinician should ask what the patient is expecting from the appointment (consultation)
2. Gathering information	The clinician should ask the patient how their fracture affected them
3. Considering therapeutic options	The clinician should, if drug treatment (medicine) is needed, offer a tablet bisphosphonate first
4. Eliciting patient perceptions	The clinician should ask the patient what they know about osteoporosis and fractures
5. Establishing shared decision-making preferences	The clinician should establish what involvement in decision-making the patient would like
6. Sharing information about condition	The clinician should show and explain the bone density scan results
7. Sharing information about treatment—lifestyle and drugs	The clinician should explain common or severe side effects
8. Checking understanding and summarise	The clinician should check whether the patient knows the benefits and risks
9. Signposting next steps	The clinician should give the patient information about local support groups and services

Clinical vignettes orientated the participant to the stage of the consultation and provided context to statements that only applied to patients with particular characteristics, e.g. a patient with bone mineral density in the osteopenic range or a patient declining treatment.

### Data collection and analysis

Consent was captured on the first page of the online survey. Participants that provided consent to Round 1 were emailed Rounds 2 and 3 by the study team. One reminder to complete the survey was sent at approximately 2 weeks for each survey round. Invitations were sent to 193 patients/carers (via the ROS supporters network) and 194 clinicians (176 via the ROS mailing lists and 18 by the study team to their clinical networks), anticipating an approximate 40% response rate to Round 1, and further drop out at Rounds 2 and 3. Our aim was to achieve an approximate final sample of 15 patients/carers and 15 clinician responders to Round 3.

#### Round 1

Participants were asked to rate their perception of the importance of each statement on a 5-point Likert scale, ranging from 5 ‘very important, should definitely be included’, to 1 ‘not important, should definitely not be included’. Participants also had the opportunity to indicate if the statement was unclear or to add additional statements for the consultation in free-text boxes.

Mean response scores were calculated for patients/carers and clinicians separately and combined. Statements with a combined mean score of less than 4 (important, should probably be included) were removed after Round 1. Statements that scored less than 4 by either patients/carers or clinicians were reviewed by a sub-group of the study team (CJ, ZP, LB), with two PAG members, to consider whether amendment was required [33]. This discussion was informed by underpinning theory, other emergent findings in iFraP-D (e.g. focus groups), PAG experiences and views, explanatory Delphi free-text comments, whether the item was scored low by patients/carers or clinicians and responses to other similar items in the Delphi. Statements were amended by changes to the contextual vignette or rewording the statement.

Statements that scored equal to or greater than 4 by clinicians and patients/carers progressed to Round 2. Statements that were indicated or described as unclear in free-text underwent minor rewording, with the PAG.

#### Round 2

Round 2 included statements that had progressed from Round 1, and new statements from free-text Delphi statements, and from iFraP-D emerging findings, as above. In the Round 2 survey, participants were shown mean scores of importance from Round 1 and asked to re-rate their perception of the importance of each statement on the same 5-point Likert scale. Free-text boxes were included for participants to give reasons or state anything that was unclear.

Mean response scores were calculated for patients/carers and clinicians separately and combined. Mean combined scores were ranked, with statements in the top 3 of 4 quartiles progressing to Round 3, as a commonly used Delphi analytical approach [34]. A small number of statements were deemed ‘considerations’ rather than tasks, which would not make sense with the amended survey in Round 3 (e.g. ‘the discussion about the benefits and risks of osteoporosis medicines is best undertaken face-to-face’). For these statements, consensus was accepted at Round 2 instead of Round 3. Statements with a patient/carer and/or clinician score in the lowest quartile were reviewed by the study sub-group and PAG members, as to whether the statement should be removed, proceed or be amended, informed by the considerations described in Round 1.

#### Round 3

Round 3 included statements that had progressed from Round 2, but no new statements. Unlike Rounds 1 and 2, in Round 3, participants were asked whether the statement was essential in a time-limited consultation of 30 min to reflect the typical length of FLS appointments. Participants rated each statement as ‘essential’ or ‘optional’.

Combined patient/carer and clinician percentage agreement was calculated as the percentage of participants rating each statement as ‘essential’. Statements were ranked according to combined percentage agreement. The study sub-group met to review the ranking and consider the appropriate threshold for essential vs optional tasks, which was not defined a priori. Tasks falling below the threshold were further reviewed with the broader study team, including experts in medicine adherence and health literacy. The purpose of this review was to consider if there was appropriate theoretical justification to re-consider any of the optional statements, as essential, in accordance with previous Delphi studies that involved discussion groups to provide comment on the inclusion or exclusion of statements [35, 36].

Finally, the essential statements were refined into a shorter list by the study team, based on the nature of the task (e.g. explaining, asking, considering) and stage of the consultation.

## Results

**Fig. 2:**
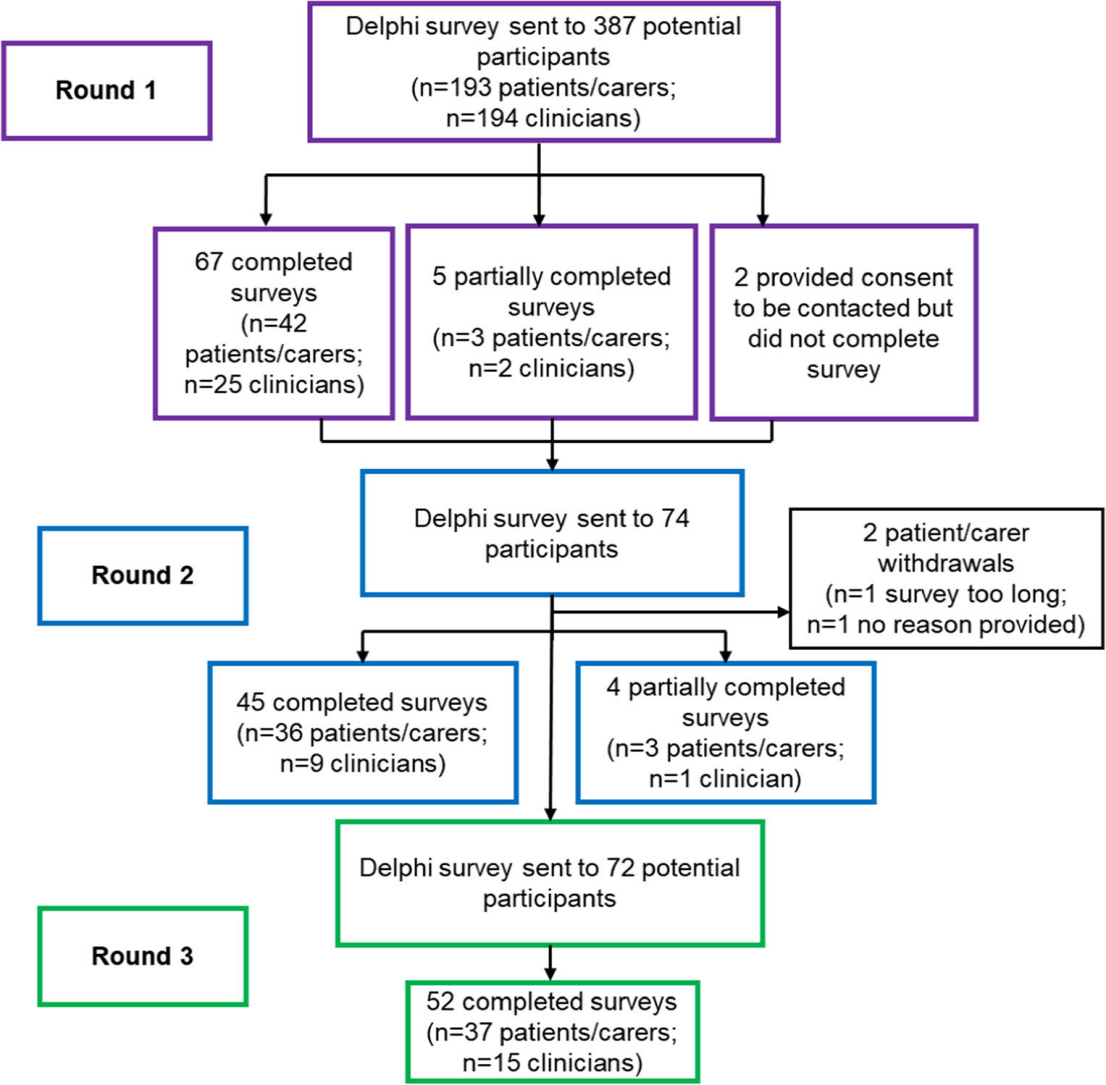
Recruitment flowchart

Seventy-two patients/carers and clinicians responded in Round 1 (23% patient/carer response rate; 14% clinician response rate), which reduced to 49 and 52 in Rounds 2 and 3, respectively (Fig. 2). Characteristics of participants in each round in Table 2 show that there were more patient/carer participants than clinicians in all three rounds (45, 39 and 37, compared with 27, 10 and 15, respectively). The majority of patients/carers were female, with the largest proportion aged between 71 and 80 years old, irrespective of survey round. All clinicians were female and most were nurses or AHPs.

**Table 2:** Participant characteristics

Patient/carer			
	Round 1	Round 2	Round 3
Total *n*	45	39	37
Gender female *n* (%)	41 (91)	35 (90)	33 (89)
Age			
21–30	0 (0)	0 (0)	0 (0)
31–40	1 (2)	1 (3)	1 (3)
41–50	0 (0)	0 (0)	0 (0)
51–60	2 (4)	3 (8)*	1 (3)
61–70	16 (36)	14 (36)	15 (41)
71–80	21 (47)	15 (39)	16 (43)
81–90	5 (11)	6 (15)	4 (11)
Clinician			
	Round 1	Round 2	Round 3
Total *n*	27	10	15
Gender female *n* (%)	27 (100)	10 (100)	15 (100)
Age			
21–30	1 (4)	0 (0)	1 (7)
31–40	4 (15)	1 (10)	2 (13)
41–50	8 (30)	4 (40)	3 (20)
51–60	9 (33)	2 (20)	4 (27)
61–70	4 (15)	2 (20)	3 (20)
71–80	0 (0)	0 (0)	1 (7)*
81–90	1 (4)	1 (10)	1 (7)
Occupation			
Allied health professional	1 (4)	0 (0)	1 (7)
FLS nurse	10 (37)	4 (40)	5 (33)
Metabolic bone specialist	1 (4)	1 (10)	1 (7)
OP specialist nurse	10 (37)	3 (30)	3 (20)
Rheumatologist	1 (4)	0 (0)	1 (7)
Other	4 (15)	2 (20)	4 (27)

*n = 2 participants self-reported as a patient or clinician differently in Delphi survey rounds

*FLS* Fracture Liaison Service, *OP* osteoporosis

A total of 122 statements were considered, each describing clinician decision-making tasks and considerations. In Round 2, seven statements were accepted as reaching consensus. In Round 3, combined percentage agreement scores were ranked, with scores ranging from 33 to 100%. The study sub-group set the threshold for inclusion as essential in a time-limited consultation as > 75% agreement. Sixty-nine (78% of Round 3 statements) statements in Round 3 were rated over this threshold and 19 (22%) statements fell below. Of these 19 statements, five were reconsidered as essential by the study team, with theoretical justification (detailed below). The flow of statements and decision-making is detailed in Supplementary Figure 1. The final statements of essential (*n* = 81) and optional (*n* = 14) consultation content and their final Round 3 percentage agreement scores are detailed in Table 3, with the 18 refined statements describing essential consultation tasks in Table 4. Scoring from Rounds 1–2 is available in Supplementary Table 2.

**Table 3:** Statements describing tasks classified as essential or optional

Statement	Round 3		
	% of participants agreeing that each statement was ‘essential in a time-limited consultation’ ≤7 5% italics NA = not applicable		
	Patient/carer agreement	Clinician agreement	Combined agreement
Stage 1: Greeting/introduction			
The clinician should...			
find out what the patient is expecting from the appointment (consultation)	*51%*	80%	*60%*^**†**^
explain to their patient that the aim of the appointment is to think about what steps they could take to improve bone health and try and prevent further broken bones	97%	100%	98%
explain that the aim is also to investigate whether the patient has osteoporosis, or weaker bones, that may be more likely to break after a minor trip or fall	92%	100%	94%
explain that the patient’s risk of breaking bones (fracture risk) in the future can be estimated	76%	87%	79%
tell the patient the limitations of estimating her risk of breaking a bone (fracture risk)*	*54%*	80%	*62%*
Stage 2: Gathering information			
The clinician should ask the patient…			
about their general health	92%	94%	92%
how the fracture impacted on their life	95%	*67%*	87%
about their risk factors for breaking a bone (fracture) which may include smoking, family history, previous fractures, alcohol, medical conditions, medications, etc.	97%	100%	98%
how their broken wrist happened	76%	100%	83%
questions to find out if the patient is at risk of falls	81%	100%	87%
about their other health conditions to identify causes of osteoporosis	87%	100%	90%
about their other health conditions to find out which medicines might be unsuitable	97%	93%	96%
if they have had back pain, or got shorter (height loss) (signs that they may have had fractures in their spine)	87%	87%	87%
questions about their diet and calcium intake	92%	100%	94%
The clinician should…			
observes the patient’s spine to look for signs of fractures (broken bones) or curvature*	76%	*60%*	*71%*
if appropriate, recommend and arrange a scan to assess the patient's bone density (strength), which will diagnose osteoporosis, if it is present	100%	100%	100%
tell the patient why the scan is being recommended, what the scan involves and how it will affect them	87%	100%	90%
arrange further imaging (such as x-rays and other tests) of the spine to look for broken bones, if appropriate	84%	*73%*	81%
use a website based scoring system (e.g. FRAX) to estimate the patient’s individual risk of breaking a bone (fracture)*	*68%*	87%	*73%*
if appropriate, recommend and arrange blood tests to rule out conditions that can make broken bones and/or osteoporosis more likely	81%	100%	87%
Stage 3: Considering therapeutic options			
The clinician should…			
use national guidelines (recommendations and guidance based on evidence) to decide which patients should be offered drug treatment (medicine) to prevent further fractures (broken bones)	N/A	N/A	N/A
be aware, and take into account, the circumstances in which the estimated fracture risk may be less accurate (e.g. for patients on high-dose steroids)	N/A	N/A	N/A
not offer tablet bisphosphonate medicines to patients who have existing problems swallowing, have severe indigestion or can’t take tablets	N/A	N/A	N/A
not offer tablet bisphosphonate medicines to patients who have memory problems (e.g. dementia), unless they have support with taking medicines	N/A	N/A	N/A
Stage 4: Eliciting patient perceptions			
The clinician should ask the patient...			
what they know about osteoporosis and fractures	84%	93%	87%
about their views on the strength of their bones*	*16%*	*73%*	*33%*
their views of prescription medicine generally*	*35%*	*53%*	*40%*
if they have any concerns generally, or if anything is on their mind	81%	80%	81%
their knowledge, views and preferences about osteoporosis medicines (drug treatments)	84%	80%	83%
how important maintaining independence is to them	81%	*67%*	77%
Stage 5: Establishing shared decision-making preferences			
The clinician should...			
establish what involvement the patient would like to have in making decisions about medicines	*68%*	87%	*73%*^**†**^
ask if the patient would like to discuss medicine or lifestyle approaches first*	*62%*	*53%*	*60%*
Stage 6: Sharing information about the condition			
The clinician should explain to the patient...			
that osteoporosis means bones are weaker and may be likely to break (fracture) after a minor bump or fall	100%	100%	100%
that osteoporosis does not give you physical symptoms (e.g. pain) unless you have broken a bone	81%	93%	85%
that keeping up a healthy lifestyle (not smoking, regular exercise) is important in maintaining bone strength and health	97%	100%	98%
that if osteoporosis medication is taken regularly it will lower the risk of breaking bones in the future	97%	100%	98%
that their bone density scan results are only part of a picture of their bone strength	87%	93%	89%
that they are at increased risk of breaking bones, using simple numbers (e.g. they have a 30 in 100 chance of breaking a bone over the next 10 years)	84%	100%	89%
that finding osteoporosis is a good thing because we can do something about it	89%	100%	92%
what risk factors they may have for weaker bones	81%	100%	87%
that people with osteoporosis are more likely to break bones such as their wrist, hip or bones in the spine	95%	100%	96%
that spinal fractures happen when the bone squashes down and may cause pain and curving of the spine	81%	80%	81%
The clinician should...			
ask the patient what they already know about how future broken bones could affect their life*	*35%*	*67%*	*44%*
use pictures or models to show how the condition affects the bone*	*57%*	*60%*	*58%*
show and explain the bone density scan results	95%	100%	96%
Stage 7: Sharing information about treatment—lifestyle and drugs			
The clinician should explain…			
how much the risk of broken bones is lowered with medicine, using simple numbers and pictures	*73%*	80%	*75%*^**†**^
that tablet medicine is usually recommended first, e.g. oral bisphosphonates	89%	93%	90%
that this medicine is recommended for osteoporosis or people with high fracture risk	100%	87%	96%
the aims and benefits of medicine, i.e. to strengthen bones and lower the chance of future broken bones	81%	100%	87%
that osteoporosis medicine does not make you feel better, and it is not possible to ‘feel’ stronger bones	*62%*	87%	*69%*^**†**^
what is involved in taking the medicine, including how long it will be taken for	100%	100%	100%
common or severe side effects	92%	100%	94%
that diet and physical activity are important in strengthening bone and have a complementary effect to medicines	100%	100%	100%
that diet, physical activity and supplements cannot be viewed as a substitute for medicines as we do not know that they work well enough to lower the risk of broken bones	76%	100%	83%
that medicines maintain bone strength and stop it from getting worse	81%	100%	87%
that osteoporosis medicines play an important role in maintaining independence and protecting your spine	78%	100%	85%
The clinician should…			
before discussing medicines, ask the patient if they have any concerns about their dental health*	*68%*	87%	*73%*
ask the patient what they knows about how lifestyle affects bone health*	*43%*	*67%*	*50%*
recommend calcium and/or vitamin D supplements as appropriate	100%	100%	100%
give general advice about avoiding falls if appropriate	78%	100%	85%
be able to discuss the benefits and risks of oral and intravenous bisphosphonates and denosumab injection	N/A	N/A	N/A
discuss the choice of medicines with the patient in this appointment	84%	87%	85%
outline the risks and benefits of injectable medicines in this appointment, so that the patient can make an informed decision about how they want to proceed	100%	100%	100%
give advice about stopping smoking and lowering alcohol intake (if appropriate)	81%	100%	87%
General			
The first discussion about the benefits and risks of osteoporosis medicines is best undertaken in the Fracture Liaison Service appointment	N/A	N/A	N/A
The discussion about the benefits and risks of osteoporosis medicines is best undertaken face-to-face	N/A	N/A	N/A
Stage 8: Checking understanding and summarise			
The clinician should check whether the patient…			
understands what the medicine will achieve	89%	93%	90%
feels sure about the best choice of drug treatment (medicine)	78%	93%	83%
knows the benefits and risks	*70%*	93%	77%
is clear about which benefits and risks matter most to them	*70%*	93%	77%
has enough support to make an informed decision about osteoporosis drug treatment (medicine)	81%	87%	83%
is happy to take the recommended option, prefers not to take it or if they are still unsure	97%	100%	98%
feels that the recommended medicine is relevant to them to meet their goals	*46%*	*73%*	*54%*^**†**^
The clinician should…			
accept the patient may have different views on risks and benefits of medicines	87%	100%	90%
check the patient’s knowledge of osteoporosis*	76%	*73%*	*75%*
check whether the patient has any concerns about the recommended medicine	92%	93%	92%
Stage 9: Discussing next steps			
The clinician should…			
explain what to do if the patient misses a dose of their medication	89%	100%	92%
explain how medication effectiveness is measured/monitored	81%	93%	85%
outline what will happen next in terms of follow-up (i.e. the patient will receive a telephone call follow-up to find out how they are getting on with the medicine, and when this will be	100%	100%	100%
if appropriate, refer the patient on to other services, e.g. falls prevention clinic	89%	100%	92%
ask the patient what questions they have	89%	93%	90%
offer advice/onward referral if the patient has concerns about fracture symptoms, such as pain	87%	93%	89%
give the patient written information about osteoporosis	87%	100%	90%
give the patient a written copy of their individualised fracture risk, and risks and benefits of drug treatment	84%	*67%*	79%
send the patient's GP a written copy of their individualised fracture risk, and risks and benefits of drug treatment	95%	93%	94%
offer a further additional telephone consultation to review in 1–2 weeks time	78%	80%	79%
arrange a standard follow-up call (in 1–2 months time)*	76%	*73%*	*75%*
give the patient information to show their dentist if prescribed bisphosphonates	76%	87%	79%
suggest that the patient considers the information and then rings a patient helpline (e.g. the Royal Osteoporosis Society helpline/a local helpline) to discuss further	95%	*73%*	89%
give contact details for where the patient can get hold of high quality information and support (e.g. the Royal Osteoporosis Society)	100%	100%	100%
explain what information the GP will receive and when*	*65%*	80%	*69%*
ask the patient how they would like to receive further information (e.g. paper, by text, website)*	76%	*73%*	*75%*
explain who to contact in case of questions	87%	100%	90%
respect the patient's decision, and continue with providing written information, further contact details in case of questions and explain about the communications the GP will receive.	100%	100%	100%
give the patient information about local support groups and services	89%	*67%*	83%

*Optional statements

^†^Deemed essential, with theoretical justification, following review by study team and experts, despite being below threshold

*N/A* not applicable, not included in Round 3 survey (consensus achieved at Round 2). For Round 1–2 results, see Supplementary Table 2

**Table 4:** FLS consultation recommendations

Consultation stage	FLS clinician should:	Additional information
Greeting/introduction	1. Explain the aim of the appointment* and find out what the patient is expecting from the appointment	*Aim includes investigating for osteoporosis, estimating risk of fracture and considering steps to improve bone health/ reduce chance of further fractures
Gathering information	2. Take a history, including information about the fracture*, medical history**, fracture risk factors***, falls and lifestyle behaviours****	*Including how the fracture happened and impact on life **To identify causes of osteoporosis and contraindications to medicines ***Such as smoking, family history, previous fractures, alcohol, medical conditions, medications etc., back pain and height loss (signs of vertebral fracture) ****General health and diet and calcium intake
	3. Recommend, arrange and explain DXA*, spinal imaging and blood tests if appropriate	*Including why DXA is recommended, what it involves and how it will affect them
Considering therapeutic options	4. Use national guidelines to decide which patients should be offered drug treatment*	*While being aware of circumstances where fracture risk may be underestimated. Do not offer oral bisphosphonates to those with severe indigestion, problems swallowing or memory problems unless they have support with taking medicines
Eliciting patient perceptions	5. Ask the patient what they already know and think about osteoporosis, osteoporosis medicines and their concerns and how important maintaining independence is	
Establishing SDM preferences	6. Establish what involvement the patient would like to have in making decisions about medicines	
Sharing information about the condition	7. Show and explain bone density results*	*In people who do not meet the densitometry definition for osteoporosis, but are recommended treatment, explain that bone density results are only part of a picture of their bone strength, but that they are at increased risk of breaking bones. Use simple numbers (e.g. they have a 30 in 100 chance of breaking a bone over the next 10 years)
	8. Explain what osteoporosis is*, the causes**, the consequences*** and how it can be controlled****	*Osteoporosis means bones are weaker and may be likely to break (fracture) after a minor bump or fall; does not give you physical symptoms (e.g. pain) unless you break a bone **Risk factors the patient has ***Mean that the patient may be more likely to break bones such as wrist, hip or bones in the spine; spinal fractures happen when the bone squashes down and may cause pain and curving of the spine ****Explain finding osteoporosis is a good thing because we can do something about it; keeping up a healthy lifestyle (not smoking, regular exercise) is important in maintaining bone strength and health; osteoporosis medication, if taken regularly will lower the risk of breaking bones in the future)
Sharing information about treatment	9. Discuss drug treatment* in a face-to-face FLS appointment, if possible, including explanation of why the treatment is recommended**, aims and benefits***, common or severe side effects, what is involved in taking the medicine and how long it will be taken for	*Oral or intravenous bisphosphonates, or denosumab **Medicine is recommended for osteoporosis or people with high fracture risk, tablet medicine is recommended first ***To strengthen bones, lower the chance of future broken bones, prevent worsening, to play an important role in maintaining independence and protecting the spine. Explain how much the risk of broken bones is lowered with medicine, using simple numbers and pictures. Explain that osteoporosis medicine does not make you feel better, and it is not possible to ‘feel’ stronger bones
	10. Discuss the role of lifestyle management*, including diet and supplements, physical activity, avoiding falls, smoking cessation and alcohol reduction if appropriate	*Explain diet and physical activity are important in strengthening bone and have a complementary effect to medicines; explain diet, physical activity and supplements cannot be viewed as a substitute for medicines as we do not know that they work well enough to lower the risk of broken bones
Checking understanding and summarise	11. Check patient understanding* and ask about any concerns	*About the benefits and risks, whether they are clear about what matters to them and how the benefits are relevant to their goals
	12. Ask* if the patient is happy to take the recommended option, prefers not to take it or if they are still unsure**	*Check they have enough support to make a decision about medicine and if they are sure about the best choice **Accept the patient may have different views on risk and benefits and respect their decision
Signposting next steps	13. Explain for patients starting treatment, how medication effectiveness is measured and monitored, what to do if a dose of medication is missed and about follow-up*	*Explain that the patient will receive a telephone call follow-up to find out how they are getting on with the medicine, and when this will be
	14. Arrange follow-up for patients who are unsure about treatment and offer helpline to discuss further	
	15. Offer advice and onward referral for fracture management and/or falls prevention if appropriate	
	16. Give the patient contact details and written information*	*About osteoporosis, individualised fracture risk, risks and benefits of treatment, information to give their dentist, advice on local and national support groups
	17. Ask what questions the patient has	
	18. Send GP a written copy of the patient’s individualised fracture risk, and of risks and benefits of drug treatment	

*Asterisks link recommendations with additional relevant information

*DXA* dual-energy X-ray absorptiometry, *GP* general practitioner, *SDM* shared decision-making

A narrative relating to the statements describing tasks which were deemed essential, optional or not relevant (removed) within each stage of the consultation is presented below, using excerpts from the participants’ free-text comments to explain the decision-making; theoretical justification of any re-classified statements is also detailed.

### Stage 1: Greeting/introduction

#### Essential

Among both patient/carer and clinician participants, there were high levels of agreement on the importance of explaining the aim of the FLS appointment and the clinician’s ability to calculate fracture risk.

A further task relating to ascertaining patient expectations of the appointment achieved 60% agreement to include in a time-limited consultation and fell below the threshold for inclusion. The study team deemed this essential, as other findings in iFraP-D illustrated that patients were often unprepared for FLS, and eliciting and addressing patient expectations is an important foundation for shared decision-making.

#### Optional

Explaining the limitations of fracture risk assessment, a task identified from clinical guidelines, received 62% agreement.

### Stage 2: Gathering information

#### Essential

Both patients/carers and clinicians had high agreement on the need for questions related to eliciting medical history, including recent and previous fracture details, diet and lifestyle, risk factors for falls, fractures and osteoporosis and signs of vertebral fracture. Further investigations including dual-energy X-ray absorptiometry (DXA), blood tests and imaging of the spine were also identified as essential, where appropriate.

#### Optional

In Round 3, ‘observe’ (reworded from earlier rounds due to concern about the term ‘examine’) the patient for signs of spinal fractures had lower agreement among clinicians (60%) than patients/carers (76%). Free-text comments revealed doubts about the applicability of physical examination to a nurse’s role and about the reliability of ‘observation’ in detecting fractures.

#### Not relevant

Asking about menopausal symptoms and conducting a physical examination as part of a falls assessment were removed in Rounds 1–2. Statements questioning the patient about what was important to them (e.g. ‘the clinician should find out what is important and what matters to the patient (e.g. hobbies, work, health, family)’) were also removed. Free-text comments questioned the relevance and specificity of these questions.

### Stage 3: Considering therapeutic options

#### Essential

Consensus was reached in Round 2 about the use of national guidelines to make clinical decisions, with the caveat that clinicians need to be aware of circumstances in which fracture risk may be underestimated by commonly used tools such as FRAX. The use of oral bisphosphonates as a first-line therapy was endorsed, except when patients had contraindications or memory problems.

### Stages 4 and 5: Eliciting patient perceptions and establishing shared decision-making preferences

#### Essential

Patients/carers and clinicians agreed that it was essential to understand the patient’s perceptions of osteoporosis and drug treatment before giving information.

Despite being rated as important by patients/carers and clinicians in Rounds 1 and 2, only 68% of patients/carers agreed that it is essential for clinicians to establish what involvement the patient would like to have in making decisions about medicines in a time-limited consultation. The study team felt this was essential due to the fundamental importance of this task in determining the nature of subsequent conversations about treatment options, using the principles of shared decision-making.

#### Optional

Asking about patients’ perceptions of the strength of their bone, their views of prescription medicine generally and their preference for discussing lifestyle or drug treatment first were all identified as optional.

### Stage 6: Sharing information about condition

#### Essential

Patients/carers and clinicians both had high agreement that it was essential for clinicians to explain what osteoporosis is, its relationship with symptoms, the individual risk factors a patient has, the consequences (in terms of broken bones and potential spinal symptoms) and the controllability of the condition. In addition, a statement added as a result of a free-text comment, about being positive that identifying osteoporosis was a good thing, so as to be able to ‘do something about it’, also achieved high (92%) agreement.

#### Optional

Using pictures or models to aid the explanation and asking what patients already know about how broken bones could affect their life were identified as optional.

#### Not relevant

In Rounds 1 and 2, statements that described the potential consequences of fracture (e.g. ‘the clinician should explain that one in ten patients with a hip fracture will die within 12 months of fracture’) were removed or amended. Free-text comments by clinicians and patients/carers described these statements as potentially gloomy and scary, with the potential to contribute to patient loss of confidence. In addition, explanatory statements related to the timing of osteoporosis (progresses slowly) and reassuring patients that there is often no cause identified were rated of relatively lower importance and removed.

### Stage 7: Sharing information about drug and lifestyle treatment

#### Essential

Consensus was gained in Round 2 that, aside from oral bisphosphonates, clinicians should be able to discuss the benefits and risks of intravenous bisphosphonate and denosumab injections and that this discussion is best conducted in a face-to-face consultation. Both patients/carers and clinicians had high agreement with statements that the discussion should include explanation of why medicine is recommended, aims and benefits, common or severe side effects, what is involved in taking the medicine and how long it will be taken for. In terms of lifestyle management, clinicians and patients/carers both rated highly discussion about diet and supplements, physical activity, avoiding falls, smoking cessation and alcohol reduction, if appropriate. However, there was less agreement among patients/carers than clinicians with the statement ‘that diet, physical activity and supplements cannot be viewed as a substitute for medicines as we do not know that they work well enough to lower the risk of broken bones’ (76% vs 100% respectively).

Two statements which fell below the threshold to include in a time-limited consultation by patients/carers and clinicians were deemed essential by the study team. First, a statement relating to communicating fracture risk improvement with medicine was deemed to be an essential component of communicating drug treatment benefit and conveying accurate drug treatment expectations. Secondly, the statement that the clinician ‘should explain that osteoporosis medicine does not make you feel better, and it is not possible to “feel” stronger bones’ only achieved 62% patient/carer agreement, yet the study team, in conjunction with expert input from a behavioural scientist, felt that setting realistic drug treatment expectations was essential to promote informed long-term adherence to medicines.

#### Optional

Asking if the patient has concerns about their dental health or knows how lifestyle affects bone health were identified as optional tasks.

#### Not relevant

Being able to discuss hormone replacement therapies and raloxifene and teriparatide injections was ranked as of relatively lower importance, with free-text statements suggesting that some respondents perceived ‘specialists’ (e.g. rheumatologists) as being better placed to discuss these treatments rather than FLS clinicians due to their unfamiliarity. Using numeric scales to ask and quantify how much side effects matter, as recommended in the Ottawa Consult Decision Aid template [28], was rated of relative lower importance and removed.

A number of statements focused on the amount of information provided to patients about the side effects of medications (e.g. ‘explain only side effects the patient is most concerned about’ and ‘explain all the side effects’). Apart from ‘explain common or severe side effects’, the statements were scored as of relatively lower importance and were removed in Rounds 1 and 2.

### Stage 8: Checking understanding and summarising

#### Essential

Tasks relating to checking patient understanding, asking about any concerns and their decision to take treatment were identified as essential.

Fifty-four percent of patients/carers and clinicians felt it was essential for the clinician to ascertain whether the patient felt the recommended medicine is relevant to their goals, meaning this task fell below the threshold. The study group felt this was an essential task, following discussion with the study behavioural scientist, due to the importance of believing drug treatment is necessary and personally relevant in fostering informed medication adherence.

#### Optional

Eliciting knowledge of osteoporosis at this stage was identified as an optional task.

#### Not relevant

In the context of a patient being unsure about treatment, the statement ‘the clinician should suggest the patient discusses further with their GP’ was removed. Free-text comments highlighted patient perceptions that GPs may have limited knowledge about osteoporosis and practical challenges of accessing their GP.

### Stage 9: Signposting next steps

#### Essential

The following end of consultation tasks were identified as essential: explanation of how medication effectiveness is measured and monitored; what to do if a dose of medication is missed; arranging follow-up and onwards referrals as appropriate; giving written information and contact details; asking what questions patients have; and sending the patient’s GP a written copy of their individualised fracture risk and risks and benefits of drug treatment. Clinicians had less agreement than patients/carers with the statements that patients should be given an individual copy of their fracture risk and treatment risks and benefits and that they should be given information about local support groups (67% vs 84% and 67% vs 89%, respectively).

#### Optional

Asking the patient in what format they would like to receive further information and explaining what information the GP would receive and when were identified as optional.

## Discussion

This Delphi exercise, informed by a wide range of evidence and theory, identified essential and optional content for the conduct of the iFraP model clinician-patient consultation within the context of FLSs. The findings have built on existing guidelines for FLSs, by focusing on the consultation, and being able to provide specific detail about the content of explanations, particularly those that help patients gain a clear understanding of their condition and treatment and address their concerns.

We have identified that consultations need to cover a core description of osteoporosis which must include a clear description of the condition and its ‘identity’, its causes, consequences and controllability [37]. The most difficult area to clarify from the results of this Delphi is related to the consequences of osteoporosis. Explaining the consequences of osteoporosis has importance for conveying the necessity for long-term medication which is important in promoting informed adherence [38]. However, patient/carer (and some clinician) responders articulated strong views in the free-text comments about statements of this nature, describing information about the potential for disability or death after fracture as fear-inducing, and at odds with the need to provide positive messages in the consultation. Our findings give practical guidance on the content of explanations for patients who do not meet bone mineral density definition of osteoporosis, but who meet clinical guidelines for use of osteoporosis medicines, an area of confusion for patients and clinicians alike [39].

The findings can be used to guide FLS clinicians in the content of their discussion about drugs. By combining statements from the NICE guidelines for medicine adherence and osteoporosis guidelines, we have addressed a gap in existing FLS Clinical Standards [4], which give information on which medications should be offered or recommended but do not give details about the content of discussion. The NICE guidelines for medicine adherence emphasise the need to take into account perceptions (e.g. necessity beliefs and concerns) and practicalities (e.g. capability and resources) that will affect individuals’ motivation and ability to start and continue with treatment [26]. Necessity beliefs relate to the extent to which an individual perceives that medicine is relevant to them and aligns with their personal goals [31]. Our results show the clinicians felt that explaining side effects (addressing concerns) and practical issues (how medicines are taken) was more important than attending to necessity beliefs, perhaps because of assumptions that patients are already convinced that they need to take their medication. However, research conducted across long-term conditions suggests that many patients harbour doubts about the necessity for regular treatment and these doubts are often linked to non-adherence. Furthermore, clinicians did not appear to highly value giving the patient a copy of their individualised fracture risk or written information on the risks and benefits of medicines (as sent to the GP), despite Clinical Standards recommending communications from the FLS are written in a style that can be understood by the patient and shared with them [19].

Our findings have revealed important insights regarding FLS clinicians’ and patients’ views of the FLS clinician role and scope of practice. The content for the Delphi was derived from four sources: osteoporosis guidelines; NICE guidelines relating to medicine adherence and patient experience; theories of shared decision-making and medicine adherence; and emerging findings from the iFraP-D study, which included views of stakeholders and PPIE members. However, of the 12 osteoporosis guidelines included, only one was targeted specifically at the FLS context [19]. The others were arguably intended for a more medical audience [22, 23]. Previous qualitative research has highlighted confusion over the roles of FLS clinicians and other clinicians involved in osteoporosis care [40, 41]. Statements relating to physical examination were rated as being of relatively low importance by clinicians, and free-text comments supported the assertion that some FLS clinicians do not perceive this as a core part of their role. The findings provide clarity on the medications that FLS clinicians feel more confident to discuss, namely oral and intravenous bisphosphonates and denosumab. Furthermore, we have identified patient/carer and FLS clinician perceptions of the distinction between the role of the FLS clinician and the primary care provider; participants indicated that detailed discussion about commonly used osteoporosis medications should take place within FLSs and not in primary care, with the exception of hormone replacement therapies (and issues related to the menopause). As this study did not include primary care clinicians, further work is needed to determine their views on these roles.

The findings give insight into difference between patient/carers and clinicians in the areas of communication perceived important. For example, while 100% of patients/carers felt explaining the importance of lifestyle management was essential, three-quarters (76%) agreed that lifestyle management cannot be viewed as a substitute for medicines. Previous qualitative research has demonstrated the high value that patients place on lifestyle management and their frustration with clinicians who may be perceived to overly focus on medications [42]. Three statements in the final essential content list were scored highly by patients but not clinicians, relating to asking about the impact of the fracture and providing written information about fracture risk and information about support groups. The reasons for clinician hesitancy in these areas should be explored. Most of the statements where patient percentage agreement that a task was essential was > 20% lower than clinicians were determined as optional, e.g. explaining the limitations of FRAX, suggesting that these tasks could be used with clinician discretion. Finally, the five statements which were reconsidered as essential by the study team were all rated higher by clinicians than patients, suggesting that patients did not highly value important elements of shared decision-making.

### Strengths and limitations

Our study has a number of strengths and weakness. Strengths include the broad range of guidelines, theory and empirical evidence that underpinned the Delphi content, which is important for designing successful interventions [43]. Furthermore, we used robust methods, adhering to methodologic criteria for reporting of Delphi studies [44]. The Delphi survey was underpinned by PPIE, with PAG members contributing to survey design, analysis and interpretation. Free-text comments provided by patient/carer participants described reworded statements as clear and understandable, recognising one of many positive outcomes associated with PAG member contribution. Despite efforts to discuss findings of Round 3 with PAG members, in the context of COVID-19 where face-to-face meetings were not possible, members did not feel confident using technologies (e.g. telephone or remote conferencing software). Despite some drop out between rounds, we managed to achieve our intended sample size, with over-recruitment of patient/carer participants. Given that we had more patient/carer participants, we had a strong patient representation; however, it was possible for decision-making to be skewed by patient/carer views. However, given many of the included items were predetermined by professional guidelines, arguably the clinical voice was strongly represented at the outset; furthermore, we took into account clinician and patient/carer ratings separately. Most clinician participants were FLS and osteoporosis specialist nurses, representative of secondary care FLSs [2] and reflecting the clinician groups that will use the iFraP intervention. The lack of other clinician groups (e.g. primary care clinicians, physician champions, endocrinologists, rheumatologists, internal medicine) participating in the survey may narrow the focus and limit generalisability of the findings; however, these other professional groups were represented in the study stakeholder group and in other iFraP-D-linked studies (e.g. focus group with primary care clinicians). Recruitment via the ROS and the use of an online survey meant that our patient/carer sample may have more digital literacy and possibly vary from the general population. However, we did recruit patient participants that are broadly representative of those attending FLSs [2], with a wide range of ages, up to the age bracket 81–90. In Round 2, ratings were generally high (all combined mean scores ≥ 4), meaning that although some items fell in the lowest quartile and were therefore removed, they were still deemed important. However, we have been careful when discussing statements that were removed, to indicate that these were rated ‘relatively’ low. The ROS FLS Clinical Standards included in our synthesis [19] have been updated since we conducted our research [4]. However, we do not feel that this would have considerably changed our Delphi content because these Clinical Standards did not change significantly in the update. However, other recently published guidance includes an algorithm to identify and treat very high-risk patients with anabolic drugs first line [45], which has implications for our findings given that we excluded discussion of teriparatide at Round 2 and may mean that knowledge of a wider range of drug treatments should be considered ‘core’ FLS activity. Finally, we incorporated an additional step after Round 3 to review optional statements and re-classify items as essential, with appropriate theoretical justification. While this could be argued to undermine the process of consensus, it is a well-accepted methodological step within Delphi to refine outputs, based on further expert views and theory [35, 36], and resulted in re-classification of less than 6% of Round 3 statements.

These findings have immediate implications for clinical practice through establishing more clearly defined tasks for FLS clinicians. Within iFraP, these findings will be synthesised with findings from other aspects of the iFraP-D study (including stakeholder workshops, focus groups with patients and clinicians, usual care survey) to guide iFraP intervention development, and specifically the design of the CDST and content of the clinician training, and training manual.

Since this study has been conducted, the COVID-19 pandemic has resulted in significant changes to clinical practice. For the most part, this does not change our recommendations, with the exception of our recommendation that discussion about risks and benefits of medicines is best undertaken face-to-face. While it may not be practicable to hold face-to-face consultations routinely for some time, further research is needed to understand the impact of consulting remotely on patient and clinical outcomes [46]. We have reported detailed recommendations to guide explanations of osteoporosis and osteoporosis medicines, but some gaps remain. Further work with stakeholders is needed to address important areas, e.g. the explanation of bone density results.

## Conclusion

This Delphi consensus exercise summarised for the first time patient/carer and clinician views regarding important content for the clinician-patient consultations in FLSs. The findings have been summarised into 18 practical recommendations which will be implemented in the iFraP consultation and also will be of wider use in training FLS clinicians and informing future iterations of FLS Clinical Standards.

## Supplementary information

ESM 1 (DOCX 225 kb)

## Data Availability

Not applicable.

## Declarations

### Disclaimer

This study presents independent research funded by the NIHR. The views expressed are those of the author(s) and not necessarily those of the National Health Service, the NIHR or the Department of Health and Social Care.
