# Supplementary material for: Developing a model Fracture Liaison Service consultation with patients, carers and clinicians: a Delphi survey to inform content of the iFraP complex consultation intervention
Source: Arch Osteoporos. 2021 Mar 24;16(1):58. doi: 10.1007/s11657-021-00913-w (PMC7989712; doi:10.1007/s11657-021-00913-w)
Supplement: Supplementary file 1 — (DOCX 225 kb) [file 11657_2021_913_MOESM1_ESM.docx]

**Supplementary Material 1.** Evidence synthesis methods

*Search strategy* - To identify osteoporosis clinical guidelines, the NHS Evidence electronic database was searched using keywords ‘fragility fracture’ and ‘osteoporosis’ and ‘guidelines’ for studies that fulfilled the eligibility criteria. The search was filtered to include Guidance, Quality Indicators, and Policy and strategy. Generic NICE guidance relating to conducting consultations was also included.

| Supplementary Table 1. Eligibility criteria for osteoporosis guideline evidence synthesis | |
| --- | --- |
| Inclusion criteria | |
| Population | Patients at risk of osteoporosis or with fragility fractures |
| Interventions | Clinician-patient consultations to assess risk and initiate/recommend treatment for fracture prevention |
| Outcomes | Assessment  Explanations  Decision-making |
| Setting | Relevant to UK primary or secondary care (e.g. UK, European or international) |
| Date | Those guidelines that have been developed, reviewed or revised within the past 10 years will be used (2009 to Feb 2019) |
| Exclusion criteria | |
| Guidelines relating to:   - managing patients already on fracture prevention treatments - disease specific conditions (e.g. screening for osteoporosis in inflammatory bowel disease) - patients with one specific fracture site (e.g. hip fracture) - steroid-induced osteoporosis - specific countries other than UK - fracture assessment and management   Guidelines related to pre-clinical and animal studies  Withdrawn guidance  The original research manuscripts underpinning the guidelines  Studies not in the English language. | |

*Selection process -* Eligible guidelines were selected on title first by one reviewer (ZP). Full texts were retrieved and assessed if the abstract provided insufficient information, again by one reviewer.

*Quality appraisal -* The quality of the included guidelines was appraised using the AGREE II tool.[34] Guidelines that score 75% or above were tagged as high quality (in line with examples given by the AGREE II developers); this quality score informed discussion in the stakeholder groups about the relevance of recommendations.

*Data extraction -* Statements and recommendations from included guidelines that were relevant to tasks for the clinician in the consultation were extracted and grouped into steps of phases of the consultation.

*Analysis -* The findings of the guideline evidence synthesis were brought together with a narrative summary, including textual description of guidelines, tabulation of recommendations and exploration of relationships between and within guidelines.

*Output -* Evidence synthesis findings were presented to the stakeholders for discussion. The synthesis output (informed by stakeholder discussion) formed the basis of the Delphi survey.


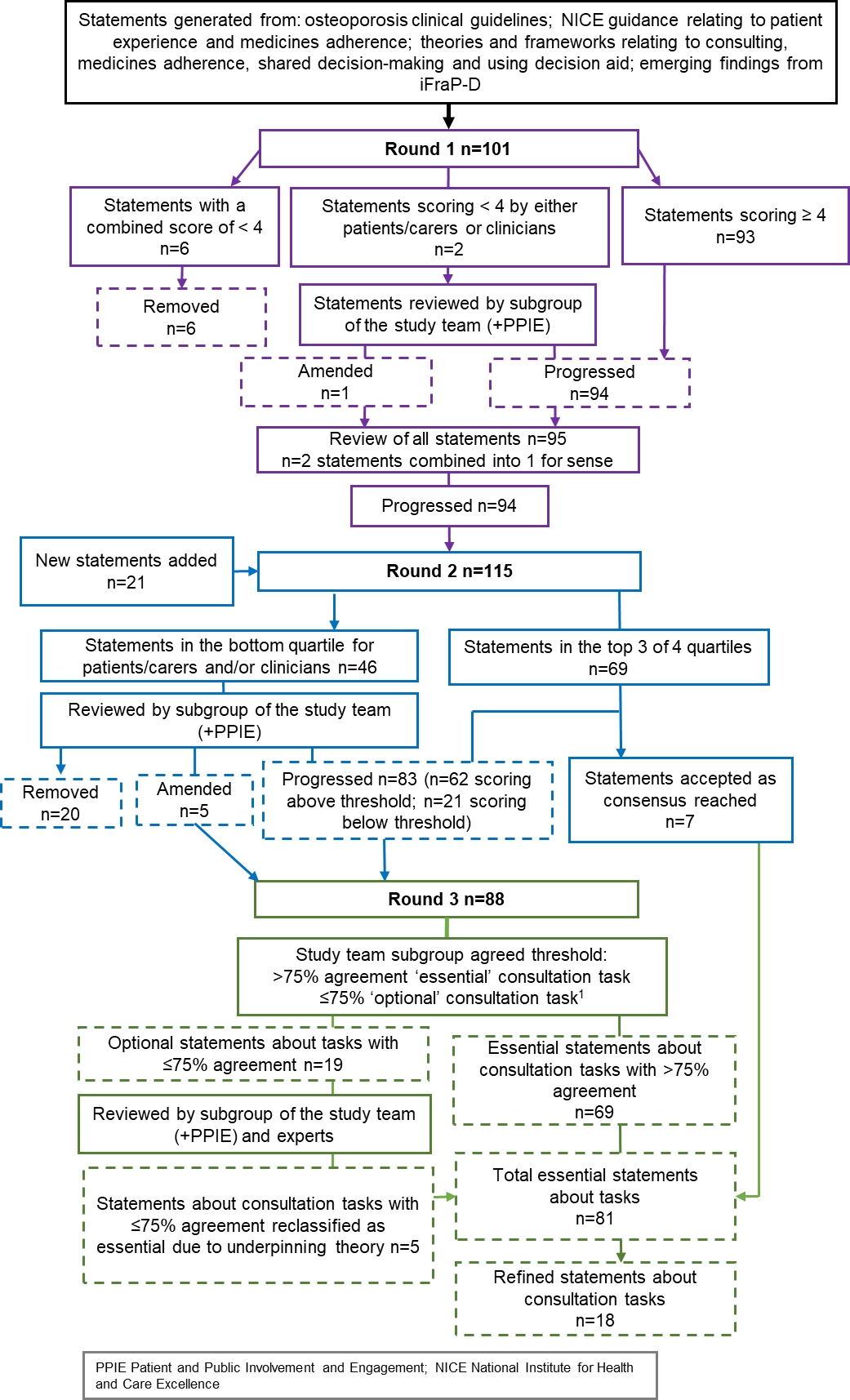


**^[[1]](#footnote-1)^**Participant percentage (%) agreement to the question ‘is this statement essential to be included in the time-limited consultation’ was calculated

**Supplementary Figure 1.** Flowchart of statements in successive Delphi survey

| **Supplementary Material 2.** Delphi survey statements with Round 1 and 2 scores | |  | | |  | | |
| --- | --- | --- | --- | --- | --- | --- | --- |
| **Delphi survey statement** | **Statement origin** | **Round 1 mean scores** | | | **Round 2 mean scores** | | |
|  |  | **Patient/carer** | **Clinician** | **Combined** | **Patient/carer** | **Clinician** | **Combined** |
| **Stage 1. Introductions/greeting** | | | | | | | |
| **The clinician should..** | | | | | | | |
| find out what the patient is expecting from the appointment (consultation) | NICE guidance relating to patient experience and medicines adherence | 4.64 | 4.81 | 4.71 | 4.59 | 4.2 | 4.51 |
| explain to their patient that the aim of the appointment is to think about what steps they could take to improve bone health and try and prevent further broken bones | Relevant theories and frameworks | 5 | 5 | 5 | 4.97 | 4.9 | 4.96 |
| explain that the aim is also to investigate whether the patient has osteoporosis, or weaker bones, that may be more likely to break after a minor trip or fall | Relevant theories and frameworks | 4.91 | 4.96 | 4.93 | 4.69 | 5 | 4.75 |
| explain that the patient's risk of breaking bones (fracture risk) in the future can be estimated | Clinical OP guidelines | 4.8 | 4.78 | 4.79 | 4.79 | 4.8 | 4.8 |
| tell the patient the limitations of estimating her risk of breaking a bone (fracture risk)* | Clinical OP guidelines | 4.71 | 4.63 | 4.68 | 4.69 | 4.2 | 4.59 |
| **Stage 2. Gathering information** | | | | | | | |
| **The clinician should ask the patient..** | | | | | | | |
| about their general health | Emerging iFraP-D findings | 4.91 | 4.85 | 4.89 | 4.92 | 4.6 | 4.86 |
| how the fracture impacted on their life | Relevant theories and frameworks | 4.84 | 4.59 | 4.75 | 4.82 | 4.5 | 4.76 |
| about their risk factors for breaking a bone (fracture) which may include smoking, family history, previous fractures, alcohol, medical conditions, medications etc. | Clinical OP guidelines | 4.93 | 4.96 | 4.94 | 4.92 | 5 | 4.94 |
| how their broken wrist happened | Clinical OP guidelines | 4.91 | 4.96 | 4.93 | 4.92 | 5 | 4.94 |
| questions to find out if the patient is at risk of falls | Clinical OP guidelines | 4.91 | 5 | 4.94 | 4.72 | 4.9 | 4.76 |
| about their other health conditions to identify causes of osteoporosis | Emerging iFraP-D findings | N/A | N/A | N/A | 4.85 | 5 | 4.88 |
| about their other health conditions to find out which medicines might be unsuitable | Emerging iFraP-D findings | N/A | N/A | N/A | 4.95 | 4.6 | 4.88 |
| if they have had back pain, or got shorter (height loss) (signs that they may have had fractures in their spine) | Clinical OP guidelines | 4.87 | 4.89 | 4.88 | 4.85 | 4.5 | 4.78 |
| questions about their diet and calcium intake | Clinical OP guidelines | 4.82 | 4.89 | 4.85 | 4.9 | 4.8 | 4.88 |
| **The clinician should...** | | | | | | | |
| observe the patient’s spine to look for signs of fractures (broken bones) or curvature* | Clinical OP guidelines | 4.56 | 3.93 | 4.32 | 4.82 | 4 | 4.65 |
| if appropriate, recommend and arrange a scan to assess the patient's bone density (strength), which will diagnose osteoporosis, if it is present | Clinical OP guidelines | 4.98 | 5 | 4.99 | 4.97 | 4.9 | 4.96 |
| tell the patient why the scan is being recommended, what the scan involves and how it will affect them | Clinical OP guidelines | 4.93 | 4.96 | 4.94 | 4.9 | 4.8 | 4.88 |
| arrange further imaging (such as x-rays and other tests) of the spine to look for broken bones, if appropriate | Clinical OP guidelines | 4.44 | 4.52 | 4.47 | 4.82 | 4.3 | 4.71 |
| use a website based scoring system (e.g. FRAX) to estimate the patient’s individual risk of breaking a bone (fracture)* | Clinical OP guidelines | 4.44 | 4.48 | 4.46 | 4.59 | 4.3 | 4.53 |
| if appropriate, recommend and arrange blood tests to rule out conditions that can make broken bones and/or osteoporosis more likely | Clinical OP guidelines | 4.69 | 4.85 | 4.75 | 4.9 | 4.7 | 4.86 |
| find out what is important and what matters to the patient (e.g. hobbies, work, health, family) | Emerging iFraP-D findings | 3.76 | 3.78 | 3.76 | N/A | N/A | N/A |
| ask what the patient's goals in life are (e.g. travelling the world)** | Emerging iFraP-D findings | 4.4 | 4.37 | 4.39 | 4.51 | 3.9 | 4.39 |
| examine the patient to see if they are at risk of falls** | Clinical OP guidelines | 4.51 | 4.22 | 4.4 | 4.49 | 4 | 4.39 |
| The clinician should, if appropriate, ask the patient if they have any symptoms of the menopause (such as flushing)** | Emerging iFraP-D findings | N/A | N/A | N/A | 4.36 | 4.4 | 4.37 |
| **Stage 3. Considering therapeutic options** | | | | | | | |
| **The clinician should..** | | | | | | | |
| use national guidelines (recommendations and guidance based on evidence) to decide which patients should be offered drug treatment (medicine) to prevent further fractures (broken bones) | Clinical OP guidelines | 4.77 | 4.92 | 4.83 | 4.79 | 4.9 | 4.82 |
| be aware, and take into account, the circumstances in which the estimated fracture risk may be less accurate (e.g. for patients on high-dose steroids) | Clinical OP guidelines | 4.77 | 4.92 | 4.83 | 4.85 | 5 | 4.88 |
| if drug treatment (medicine) is recommended, offer a tablet bisphosphonate first** | Clinical OP guidelines | 4.14 | 4.73 | 4.36 | 4.05 | 4.5 | 4.14 |
| not offer tablet bisphosphonate medicines to patients who have existing problems swallowing, have severe indigestion or can’t take tablets | Clinical OP guidelines | 4.63 | 5 | 4.77 | 4.69 | 4.9 | 4.73 |
| not offer tablet bisphosphonate medicines to patients who have memory problems (e.g. dementia), unless they have support with taking medicines | Clinical OP guidelines | 4.48 | 4.92 | 4.64 | 4.51 | 4.6 | 4.53 |
| **Stage 4. Elicit patient perceptions** | | | | | | | |
| **The clinician should ask the patient..** | | | | | | | |
| what they know about osteoporosis and fractures | Relevant theories and frameworks | 4.8 | 4.85 | 4.82 | 4.87 | 4.7 | 4.84 |
| their views on the strength of their bones* | Relevant theories and frameworks | 4.49 | 4.41 | 4.46 | 4.51 | 4.1 | 4.43 |
| how important maintaining independence is to them | Relevant theories and frameworks | 4.73 | 4.7 | 4.72 | 4.72 | 4.5 | 4.67 |
| their views of prescription medicine generally* | Relevant theories and frameworks | 4.38 | 4.52 | 4.43 | 4.51 | 4.1 | 4.43 |
| patient if they have any concerns generally, or if anything is on their mind | NICE guidance relating to patient experience and medicines adherence | 4.44 | 4.52 | 4.47 | 4.64 | 4.1 | 4.53 |
| their knowledge, views and preferences about osteoporosis medicines (drug treatments) | NICE guidance relating to patient experience and medicines adherence | 4.4 | 4.37 | 4.78 | 4.79 | 4.4 | 4.71 |
| **Stage 5. Establish shared decision making preferences** | | | | | | | |
| **The clinician should...** | | | | | | | |
| establish what involvement the patient would like to have in making decisions about medicines | NICE guidance relating to patient experience and medicines adherence | 4.79 | 4.8 | 4.79 | 4.84 | 4.56 | 4.79 |
| ask if the patient would like to discuss medicine or lifestyle approaches first* | Emerging iFraP-D findings | 4.6 | 4.77 | 4.67 | 4.55 | 4.33 | 4.51 |
| **Stage 6. Share information about condition** | | | | | | | |
| **The clinician should explain…** | | | | | | | |
| that osteoporosis means bones are weaker and may be likely to break (fracture) after a minor bump or fall | Emerging iFraP-D findings | 4.86 | 5 | 4.91 | 4.69 | 5 | 4.75 |
| that osteoporosis does not give you physical symptoms (e.g. pain) unless you have broken a bone | Emerging iFraP-D findings | 4.81 | 4.92 | 4.86 | 4.85 | 4.78 | 4.83 |
| that although the bone scan does not show osteoporosis, the patient's high fracture risk means that it is still likely they have weak bones** | NICE guidance relating to patient experience and medicines adherence | 4.4 | 4.35 | 4.38 | 4.59 | 4 | 4.48 |
| that having one broken bone makes you more likely to have another** | Emerging iFraP-D findings | N/A | N/A | N/A | 4.33 | 4.67 | 4.4 |
| that osteoporosis usually develops slowly over time** | Relevant theories and frameworks | 4.49 | 4.77 | 4.59 | 4.49 | 4.33 | 4.46 |
| that broken bones (e.g. hip fractures) may lead to loss of independence and confidence, feeling less well and having difficulty with hobbies, work and self-care** | Relevant theories and frameworks | 4.44 | 4.54 | 4.48 | 4.33 | 4.44 | 4.35 |
| that one in three patients with a hip fracture will no longer be able to live in their own home** | Relevant theories and frameworks | 3.84 | 3.81 | 3.83 | N/A | N/A | N/A |
| that keeping up a healthy lifestyle (not smoking, regular exercise) is important in maintaining bone strength and health | Clinical OP guidelines | 4.84 | 4.88 | 4.86 | 4.9 | 4.78 | 4.88 |
| that if osteoporosis medication is taken regularly it will lower the risk of breaking bones in the future | Clinical OP guidelines | 4.79 | 4.92 | 4.84 | 4.82 | 4.89 | 4.83 |
| that their bone density scan results are only part of a picture of their bone strength | Emerging iFraP-D findings | 4.69 | 4.54 | 4.63 | 4.82 | 4.67 | 4.79 |
| that they are at increased risk of breaking bones, using simple numbers (e.g. they have a 30 in 100 chance of breaking a bone over the next 10 years) | NICE guidance relating to patient experience and medicines adherence | 4.33 | 4.65 | 4.46 | 4.64 | 3.89 | 4.5 |
| that one in ten patients with a hip fracture will die within 12 months of fracture** | Relevant theories and frameworks | 3.37 | 3.5 | 3.42 | N/A | N/A | N/A |
| their individual risk of breaking any major bone over the next 10 years** | Relevant theories and frameworks | 4.14 | 4.31 | 4.2 | 4.24 | 4 | 4.19 |
| their individual risk of breaking a hip over the next 10 years** | Relevant theories and frameworks | 4.12 | 4.27 | 4.17 | 4.15 | 4.11 | 4.15 |
| that finding osteoporosis is a good thing because we can do something about it | Emerging iFraP-D findings | N/A | N/A | N/A | 4.74 | 4.56 | 4.71 |
| what risk factors they may have for weaker bones | Emerging iFraP-D findings | N/A | N/A | N/A | 4.77 | 4.67 | 4.75 |
| that people with osteoporosis are more likely to break bones such as their wrist, hip or bones in the spine | Emerging iFraP-D findings | N/A | N/A | N/A | 4.82 | 4.67 | 4.79 |
| that spinal fractures happen when the bone squashes down and may cause pain and curving of the spine | Emerging iFraP-D findings | N/A | N/A | N/A | 4.54 | 4.44 | 4.52 |
| **The clinician should..** | | | | | | | |
| reassure that osteoporosis is common and there is no single cause** | Emerging iFraP-D findings | 4.56 | 4.77 | 4.64 | 4.46 | 4.44 | 4.46 |
| ask the patient what they already know about how future broken bones could affect their life* | Emerging iFraP-D findings | 4.35 | 4.54 | 4.42 | 4.08 | 3.89 | 4.04 |
| use pictures or models to show how the condition affects the bone* | Clinical OP guidelines | 4.51 | 4.65 | 4.57 | 4.59 | 4.56 | 4.58 |
| show and explain the bone density scan results | Emerging iFraP-D findings | 4.77 | 4.81 | 4.78 | 4.59 | 4.56 | 4.58 |
|  |  |  |  |  |  |  |  |
| **Stage 7. Share information about treatment options** | | | | | | | |
| **The clinician should explain..** | | | | | | | |
| how much the risk of broken bones is lowered with medicine, using simple numbers and pictures | Relevant theories and frameworks | 3.88 | 4.31 | 4.04 | 4.1 | 4 | 4.08 |
| that tablet medicine is usually recommended first e.g. oral bisphosphonates | Clinical OP guidelines | 4.7 | 4.85 | 4.75 | 4.76 | 4.67 | 4.74 |
| that this medicine is recommended for osteoporosis or people with high fracture risk | Emerging iFraP-D findings | 4.81 | 4.96 | 4.87 | 4.84 | 4.56 | 4.79 |
| the aims and benefits of medicine, i.e. to strengthen bones and lower the chance of future broken bones | NICE guidance relating to patient experience and medicines adherence | 4.91 | 4.96 | 4.93 | 4.89 | 4.89 | 4.89 |
| that osteoporosis medicine does not make you feel better, and it is not possible to ‘feel’ stronger bones | Relevant theories and frameworks | 4.62 | 4.85 | 4.71 | 4.61 | 4.11 | 4.51 |
| what is involved in taking the medicine, including how long it will be taken for | Clinical OP guidelines | 4.88 | 5 | 4.93 | 4.92 | 4.78 | 4.89 |
| all the side effects of this medicine** | Clinical OP guidelines | 4.4 | 4.16 | 4.31 | 4.32 | 3.89 | 4.23 |
| only side effects the patient is most concerned about** | Clinical OP guidelines | 4.03 | 3.6 | 3.88 | N/A | N/A | N/A |
| common or severe side effects | Clinical OP guidelines | 4.17 | 4.17 | 4.17 | 4.82 | 4.67 | 4.79 |
| **The clinician should explain..** | | | | | | | |
| that diet and physical activity are important in strengthening bone and have a complementary effect to medicines | Clinical OP guidelines | 4.86 | 4.92 | 4.88 | 4.97 | 4.89 | 4.96 |
| that diet, physical activity and supplements cannot be viewed as a substitute for medicines as we do not know that they work well enough to lower the risk of broken bones | Emerging iFraP-D findings | 4.28 | 4.65 | 4.42 | 4.53 | 4.33 | 4.49 |
| that medicines maintain bone strength and stop it from getting worse | Emerging iFraP-D findings | N/A | N/A | N/A | 4.79 | 4.78 | 4.79 |
| that osteoporosis medicines lower the risk of future broken bones in the same way that statins lower the risk of future heart attacks** | Emerging iFraP-D findings | N/A | N/A | N/A | 4.39 | 3.89 | 4.3 |
| that osteoporosis medicines play an important role in maintaining independence and protecting your spine | Emerging iFraP-D findings | N/A | N/A | N/A | 4.68 | 4.33 | 4.62 |
| **The clinician should…** | | | | | | | |
| recommend calcium and/or vitamin D supplements as appropriate | Clinical OP guidelines | 4.7 | 4.96 | 4.8 | 4.87 | 4.89 | 4.87 |
| give general advice about avoiding falls if appropriate | Clinical OP guidelines | 4.7 | 4.96 | 4.8 | 4.74 | 4.67 | 4.72 |
| be able to discuss the benefits and risks of..  1. raloxifene  2. denosumab injection  3. bisphosphonate given intravenously  4. teriparatide injection  5. Hormone Replacement Therapy (HRT) | Clinical OP guidelines | 4.53 | 4.15 | 4.39 | 1. 4.55 2. 4.58 3. 4.53 4. 4.47 5. 4.53 | 1. 4.11 2. 4.67 3. 4.44 4. 4.22 5. 3.78 | 1. 4.47 2. 4.6 3. 4.51 4. 4.43 5. 4.38 |
| discuss the choice of medicines with the patient in this appointment | Clinical OP guidelines | 4.6 | 4.65 | 4.62 | 4.53 | 4.78 | 4.57 |
| refer the patient to their GP or a specialist to discuss other medicines** | Emerging iFraP-D findings | 4.26 | 4.25 | 4.26 | 4.34 | 4.22 | 4.32 |
| offer a further appointment to discuss injectable medicines, and explain why the appointment is being offered ^†^ | Emerging iFraP-D findings | 4.65 | 4.63 | 4.64 | N/A | N/A | N/A |
| use a combination of pictures and spoken frequencies (e.g. 1 in 1000 people) when discussing side effects to show how common (or rare) the side effects are** | Relevant theories and frameworks | 4.16 | 4.35 | 4.23 | 4.45 | 4 | 4.36 |
| not recommend medicines that are not approved (licensed) for men unless no alternative exists | Clinical OP guidelines | 4.14 | 4.42 | 4.24 | 4.28 | 3.78 | 4.19 |
| outline the risks and benefits of injectable medicines in this appointment, so that the patient can make an informed decision about how they want to proceed ^†^ | Emerging iFraP-D findings | 4.72 | 4.72 | 4.72 | 4.61 | 4.89 | 4.66 |
| give the patient written information about all side effects** | Emerging iFraP-D findings | 4.28 | 4.46 | 4.35 | 4.29 | 4.56 | 4.34 |
| offer a choice of alternative medicines** | Emerging iFraP-D findings | N/A | N/A | N/A | 4.39 | 4.44 | 4.4 |
| give advice about stopping smoking and lowering alcohol intake (if appropriate) | Emerging iFraP-D findings | N/A | N/A | N/A | 4.71 | 4.67 | 4.7 |
| ask the patient what they know about how lifestyle affects bone health* | Emerging iFraP-D findings | 4.73 | 4.7 | 4.83 | 4.87 | 4.6 | 4.82 |
| ask the patient if they have any concerns about their dental health* | Emerging iFraP-D findings | 4.38 | 4.52 | 4.47 | 4.56 | 4 | 4.45 |
| ask the patient how much lowering their risk of broken bones (e.g. hip fracture) matters to them, using a simple scale (e.g. It matters a great deal…it does not matter)** | Relevant theories and frameworks | 4.21 | 3.96 | 4.12 | 4.32 | 4.22 | 4.3 |
| ask the patient how much each side effects matters to them using a simple scale (e.g. it matters a great deal…it does not matter)** | Relevant theories and frameworks | 3.93 | 3.76 | 3.87 | N/A | N/A | N/A |
| **General** | | | | | | | |
| The first discussion about the benefits and risks of osteoporosis medicines can occur before the patient blood test results are known** | Emerging iFraP-D findings | N/A | N/A | N/A | 4.03 | 4.22 | 4.06 |
| The first discussion about the benefits and risks of osteoporosis medicines is best undertaken in the Fracture Liaison Service appointment | Emerging iFraP-D findings | N/A | N/A | N/A | 4.74 | 4.78 | 4.74 |
| The discussion about the benefits and risks of osteoporosis medicines is best undertaken face to face | Emerging iFraP-D findings | N/A | N/A | N/A | 4.89 | 4.78 | 4.87 |
| **Stage 8. Check understanding and summarise** | | | | | | | |
| **The clinician should check whether the patient..** | | | | | | | |
| understands what the medicine will achieve | NICE guidance relating to patient experience and medicines adherence | 4.86 | 4.92 | 4.88 | 4.82 | 4.89 | 4.83 |
| feels sure about the best choice of drug treatment (medicine) | Relevant theories and frameworks | 4.69 | 4.88 | 4.76 | 4.76 | 4.56 | 4.72 |
| knows the benefits and risks | Relevant theories and frameworks | 4.83 | 4.88 | 4.85 | 4.84 | 4.78 | 4.83 |
| is clear about which benefits and risks matter most to them | Relevant theories and frameworks | 4.67 | 4.68 | 4.67 | 4.76 | 4.67 | 4.74 |
| has enough support to make an informed decision about osteoporosis drug treatment (medicine) | Relevant theories and frameworks | 4.81 | 4.92 | 4.85 | 4.84 | 4.56 | 4.79 |
| is happy to take the recommended option, prefers not to take it or if they are still unsure | Relevant theories and frameworks | 4.78 | 4.88 | 4.82 | 4.89 | 4.78 | 4.87 |
| has any concerns about the recommended medicine | Relevant theories and frameworks | N/A | N/A | N/A | 4.84 | 4.89 | 4.85 |
| feels that the recommended medicine is relevant to them to meet their goals | Relevant theories and frameworks | N/A | N/A | N/A | 4.55 | 4.33 | 4.51 |
| **The clinician should...** | | | | | | | |
| check if the patient is unsure about drug treatment, the clinician should suggest that they discuss further with their GP** | Emerging iFraP-D findings | 3.76 | 4.44 | 3.99 | N/A | N/A | N/A |
| suggest that the patient considers the information and then rings a patient helpline (e.g. the Royal Osteoporosis Society helpline/a local helpline) to discuss further | Clinical OP guidelines | 4.67 | 4.56 | 4.63 | 4.78 | 4.44 | 4.72 |
| offer a further additional telephone consultation to review in 1-2 weeks time | Emerging iFraP-D findings | 4.36 | 4.68 | 4.48 | 4.65 | 4.44 | 4.61 |
| arrange a standard follow-up call (in 1-2 months’ time)* | Clinical OP guidelines | 4.79 | 4.68 | 4.75 | 4.59 | 4.33 | 4.54 |
| find out the patient's knowledge of osteoporosis* | Relevant theories and frameworks | N/A | N/A | N/A | 4.89 | 4.78 | 4.87 |
| accept the patient may have different views on risks and benefits of medicines | NICE guidance relating to patient experience and medicines adherence | 4.65 | 4.96 | 4.77 | 4.66 | 4.67 | 4.66 |
| **Stage 9. Signpost next steps** | | | | | | | |
| **The clinician should…** | | | | | | | |
| explain what to do if the patient misses a dose of their medication | Clinical OP guidelines | 4.88 | 4.68 | 4.81 | 4.83 | 4.89 | 4.84 |
| explain how medication effectiveness is measured/monitored | Clinical OP guidelines | 4.64 | 4.8 | 4.7 | 4.75 | 4.78 | 4.76 |
| outline what will happen next in terms of follow-up (i.e. the patient will receive a telephone call follow-up to find out how they are getting on with the medicine, and when this will be | Relevant theories and frameworks | 4.79 | 4.84 | 4.81 | 4.75 | 4.89 | 4.78 |
| if appropriate, refer the patient on to other services e.g. falls prevention clinic | Clinical OP guidelines | 4.71 | 4.92 | 4.79 | 4.75 | 4.78 | 4.76 |
| ask the patient what questions they have | Relevant theories and frameworks | 4.9 | 4.92 | 4.91 | 4.94 | 4.78 | 4.91 |
| offer advice/onward referral if the patient has concerns about fracture symptoms, such as pain | Clinical OP guidelines | 4.76 | 4.88 | 4.81 | 4.83 | 4.78 | 4.82 |
| give the patient written information about osteoporosis | Clinical OP guidelines | 4.76 | 4.96 | 4.84 | 4.75 | 4.89 | 4.78 |
| give the patient a personalised written copy of the risks and benefits of drug treatment and individual fracture risk | Emerging iFraP-D findings | 4.44 | 4.68 | 4.53 | 4.58 | 4.33 | 4.53 |
| send the patient's GP a written copy of their individualised fracture risk, and risks and benefits of drug treatment | Emerging iFraP-D findings | 4.69 | 4.72 | 4.7 | 4.92 | 4.78 | 4.89 |
| explain to the patient what information the GP will receive and when* | Clinical OP guidelines | 4.55 | 4.64 | 4.58 | 4.58 | 4.67 | 4.6 |
| give the patient information to show their dentist if prescribed bisphosphonates | Clinical OP guidelines | 4.93 | 4.88 | 4.91 | 4.5 | 4.44 | 4.49 |
| give contact details for where the patient can get hold of high quality information and support (e.g. the Royal Osteoporosis Society) | Clinical OP guidelines | 4.83 | 4.96 | 4.88 | 4.92 | 4.89 | 4.91 |
| explain who to contact in case of questions | Clinical OP guidelines | 4.55 | 4.84 | 4.66 | 4.75 | 4.78 | 4.76 |
| respect the patient's decision, and continue with providing written information, further contact details in case of questions and explain about the communications the GP will receive. | NICE guidance relating to patient experience and medicines adherence | 4.8 | 4.96 | 4.86 | 4.83 | 4.89 | 4.84 |
| give the patient information about local support groups and services | Emerging iFraP-D findings | N/A | N/A | N/A | 4.75 | 4.11 | 4.62 |
| ask the patient how they would like to receive further information (e.g. paper, by text, website)* | Emerging iFraP-D findings | N/A | N/A | N/A | 4.61 | 4.33 | 4.56 |
| *Statements considered optional in Round 3  **Statements removed in Round 1 or Round 2  ^†^Two statements combined into one for sense in Round 2  OP osteoporosis, NICE National Institute for Health and Care Excellence, GP general practitioner | | | | | | | |

1. [↑](#footnote-ref-1)
